# Engineering Pore Accessibility in PVA/Cellulose Nanocrystal/Ca^2+^ Composite Aerogels for Enhanced Drug Adsorption and Sustained Release

**DOI:** 10.3390/gels12090791

**Published:** 2026-09-01

**Authors:** Zixuan Zhang, Qianqian Wu, Hua Jiang

**Affiliations:** College of Chemical Engineering, Nanjing Forestry University, Nanjing 210037, China; 8230210202@njfu.edu.cn (Z.Z.); 19952303332@163.com (Q.W.)

**Keywords:** poly (vinyl alcohol), cellulose nanocrystals, solvent exchange, drug delivery, aerogel

## Abstract

While poly (vinyl alcohol) (PVA)-based aerogels are promising porous carriers for controlled drug delivery, drying-induced pore collapse generally limits their internal pore accessibility, drug-loading capacity, and release regulation. In this study, duration-controlled tert-butanol (TBA) solvent exchange was developed as a pore-engineering strategy to regulate the structure-performance relationship of PVA/cellulose nanocrystal/Ca^2+^ composite aerogels. This strategy focuses on balancing pore preservation, liquid-accessible pore connectivity, and network densification, instead of simply increasing the total surface area. With an optimized exchange duration, the TBA6 aerogel exhibited a well-preserved porous network. Moreover, its specific surface area and porosity increased from 33.11 to 96.02 m^2^·g^−1^ and from 93.93% to 96.03%, respectively. This optimized structure enhanced salicylic acid (SA) loading, elevating the equilibrium adsorption capacity from 40.91 to 61.18 mg·g^−1^. Release kinetic analysis revealed that the Higuchi constant and the Korsmeyer-Peppas release constant decreased from 18.148 to 12.751 and from 25.070 to 17.283%·h^−1/2^, respectively, indicating a slower diffusion-regulated release process. Confocal Raman mapping further confirmed that TBA6 promoted a more homogeneous SA distribution within the aerogel matrix and enabled gradual release from the internal porous network. To sum up, controlling solvent-exchange duration is an effective strategy for tuning pore accessibility and diffusion resistance. This study provides new insight into the design of PVA-based composite aerogels for drug-loading and sustained-release applications.

## 1. Introduction

Polymer aerogels, featuring ultralow density, high porosity, and three-dimensional interconnected networks, have attracted considerable attention [1] and become promising materials for adsorption, separation, and controlled delivery applications [2]. Among them, PVA-based aerogels are particularly attractive for biomedical and drug delivery applications, attributed to their excellent biocompatibility, hydrophilicity, and facile processability [3,4]. The structural stability and functionality of PVA-based aerogels were improved through incorporation of nanofillers and ionic crosslinking strategies [5]. Particularly, cellulose nanocrystals (CNCs) were selected because their high aspect ratio, rigidity, and surface hydroxyl/carboxylate groups are beneficial for reinforcing the PVA/Ca^2+^ network. The size and dispersion state of CNCs may also influence pore formation. Specifically, well-dispersed CNCs can support a more uniform and connected network, whereas a broad size distribution or local aggregation may bring about heterogeneous pores, partial pore blocking, and increased mass-transfer resistance. The abundant hydroxyl groups of CNCs can form hydrogen bonds with PVA; the carboxylate groups introduced by ammonium persulfate oxidation can interact with Ca^2+^ and contribute to ionic coordination within the composite network. Additionally, the high aspect ratio and rigidity of CNCs assist in enhancing the hydrogel skeleton and preserving the porous structure during solvent exchange and freeze-drying. Compared with inorganic nanofillers, CNCs are renewable, biocompatible, and more compatible with hydrophilic polymer networks, thereby being suitable for constructing PVA-based aerogel carriers for drug loading and sustained release [6,7,8,9].

Despite these advances, a persistent limitation of PVA-based aerogels lies in their structural instability during drying processes [10,11]. Conventional freeze-drying typically leads to ice-crystal growth, crystallization-induced stresses, and solute redistribution during freezing, which all disturb the hydrogel network, resulting in pore collapse, shrinkage, and heterogeneous pore formation after sublimation [12]. These structural defects significantly reduce specific surface area and internal pore accessibility, thereby restricting adsorption capacity and impairing controlled release behavior [13,14,15]. Although modifications in freezing conditions, crosslinking density, and filler incorporation have been widely explored, these approaches primarily emphasize the improvement of mechanical robustness rather than the fundamental regulation of the pore accessibility-diffusion relationship governing mass transport [16,17].

Solvent exchange has been recognized as an effective strategy for reducing drying-induced structural deformation in aerogels [18]. The solvent composition, freezing behavior, and solvent distribution within the hydrogel network can be regulated by partially replacing water with TBA. This influences crystal formation, local solute redistribution, and polymer-network rearrangement during freezing. These effects contribute to better preservation of the porous structure after freeze-drying [19,20]. Among various solvents, TBA is particularly advantageous because its moderate polarity, low surface tension, and favorable crystallization behavior collectively facilitate the formation of more open and uniform pore structures during freeze-drying [21]. Furthermore, solvent exchange can improve surface area and mechanical stability [16,22,23]. Nonetheless, most reports mainly correlate solvent exchange with morphological improvement while neglecting its influence on transport-controlled functions such as adsorption kinetics and diffusion-regulated release [24].

Moreover, existing studies generally treat solvent exchange as a binary or process parameter (presence or absence), without the systematic consideration of the role of exchange duration in regulating microstructural evolution [25,26,27]. In polymeric hydrogel-derived aerogels, solvent-exchange time is expected to govern the extent of water replacement, polymer chain rearrangement, and pore-wall consolidation, enabling the determination of the balance between pore accessibility and network densification [28,29,30]. However, the structure–property relationship linking solvent-exchange duration to adsorption capacity and sustained release behavior remains insufficiently understood. Particularly, how pore accessibility and diffusion pathways evolve with solvent-exchange time, as well as how these structural changes govern drug loading and release kinetics, has not been clearly elucidated [13].

In this work, PVA/CNCs/Ca^2+^ composite aerogels with a fixed CNC content were fabricated and underwent TBA solvent exchange with controlled durations. SA was selected as a model drug to evaluate adsorption and sustained release performance. With a focus on the role of solvent-exchange duration within this fixed composite composition, the results demonstrated the effect of TBA exchange duration on the PVA/CNCs/Ca^2+^ aerogel system rather than independently isolating the contribution of CNCs compared with CNC-free aerogels. It was hypothesized in this study that solvent-exchange duration can precisely tune the internal pore structure by balancing pore preservation and network densification, allowing for the regulation of both adsorption site accessibility and diffusion resistance. Additionally, the effects of solvent-exchange duration on microstructure, specific surface area, porosity, liquid uptake behavior, mechanical stability, and mass transport properties were systematically investigated. Furthermore, a unified structure–property relationship linking solvent-exchange duration, pore accessibility, and diffusion-controlled release behavior in PVA-based composite aerogels was established by correlating structural parameters with adsorption capacity and release kinetics. This study provides new insights for the rational design of aerogel-based drug delivery systems.

## 2. Results and Discussion

### 2.1. Density and Gravimetric PBS Uptake Behavior of the Optimized Aerogels

**Figure 1:**
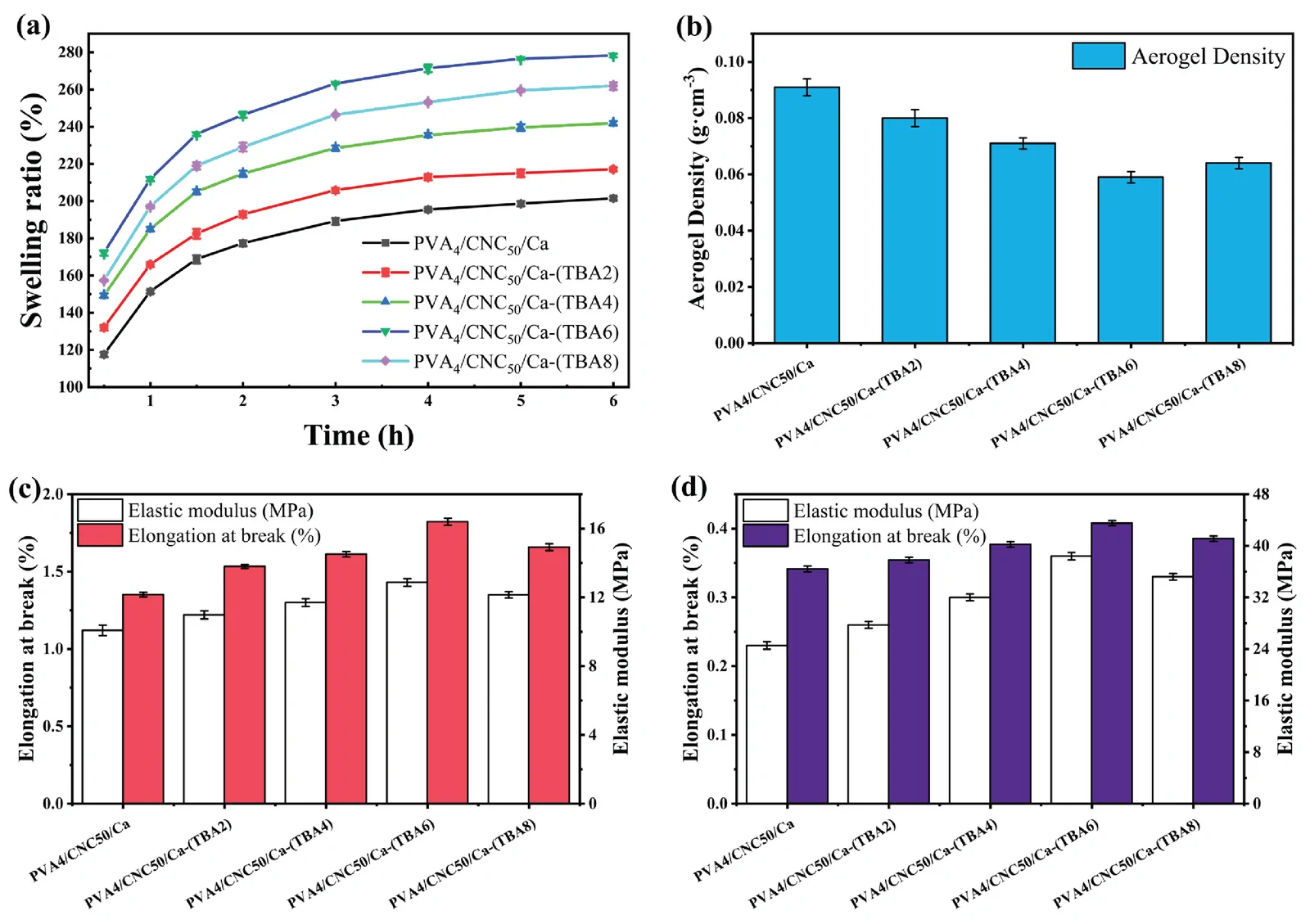
Liquid uptake behavior, density, and tensile properties of PVA_4_/CNC_50_/Ca composite aerogels after TBA solvent exchange: (**a**) time-dependent gravimetric PBS uptake ratio; (**b**) aerogel density; (**c**) elongation at break and elastic modulus of dry aerogels; (**d**) elongation at break and elastic modulus of swollen aerogels.

Figure 1 illustrates the gravimetric phosphate-buffered saline (PBS) uptake behavior, aerogel density, and tensile properties of PVA_4_/CNC_50_/Ca aerogels after different TBA exchange durations. As observed from Figure 1a, all samples absorbed PBS rapidly at the initial stage and then gradually approached swelling equilibrium. The untreated aerogel exhibited the lowest gravimetric PBS uptake ratio, and the TBA-treated samples demonstrated higher gravimetric PBS uptake capacity. Among them, TBA6 reached the highest gravimetric PBS uptake ratio and maintained a stable swelling plateau. In Figure 1b, the aerogel density decreased after TBA exchange, with TBA6 presenting the lowest density. A slight increase in density was observed for TBA8. The tensile results in Figure 1c,d reflected that both the dry and swollen aerogels retained measurable mechanical integrity. The elongation at break and elastic modulus increased after solvent exchange and reached relatively high values for TBA6, and TBA8 displayed a slight decline or no further improvement.

These results reveal that TBA solvent exchange strongly influenced the wet-state accessibility and structural stability of the PVA_4_/CNC_50_/Ca aerogels. The untreated aerogel exhibited the lowest liquid uptake and relatively high density. In other words, direct freeze-drying triggered partial shrinkage and limited the accessibility of the internal porous network. After TBA exchange, the liquid uptake significantly increased, and the density decreased, implying that solvent exchange improved preservation of the porous framework and facilitated liquid penetration into the internal pores. Among the treated samples, TBA6 displayed the highest liquid uptake and the lowest density. Thus, an appropriate exchange duration can promote pore openness without causing excessive network consolidation [19,20]. Among the treated samples, TBA6 presented the highest gravimetric PBS uptake ratio and the lowest density, suggesting that an appropriate exchange duration promoted liquid penetration into the internal pores rather than limiting PBS uptake to the external surface.

Nonetheless, the evolution from TBA6 to TBA8 underscores that prolonged solvent exchange failed to further improve the overall liquid accessibility. Although extended exchange may further refine the pore structure, the slight increase in density and the lower liquid uptake capacity of TBA8 may be consistent with partial network contraction or pore-wall densification. Such densification can restrict solvent uptake and weaken the effective accessibility of internal adsorption sites. Hence, the role of TBA exchange is to both increase porosity and regulate the balance between pore opening and network consolidation, as reflected in the swelling and density results [26,28,31]. TBA6 represents the most favorable structural state, in which the aerogel maintains an open and accessible pore network without excessive densification.

Furthermore, the improved pore accessibility of TBA6 was not achieved at the expense of network integrity according to tensile results. Both the dry and swollen aerogels retained measurable mechanical strength, confirming that the PVA/CNCs/Ca^2+^ network remained stable after solvent exchange. For drug-loading and sustained-release applications, an overly loose network may bring about excessive swelling and rapid drug diffusion, whereas an overly dense network can hinder liquid transport and reduce drug loading [15,17,32]. The close relationship between polymer-network structure and liquid-uptake behavior has also been demonstrated in cellulose-based hydrogel systems, where variations in network composition significantly affected swelling, water transport, and mechanical properties [33]. TBA6 demonstrated a favorable compromise between these two extremes. Its low density and high gravimetric PBS uptake capacity indicate enhanced pore accessibility, while its retained elastic modulus and elongation at break confirm sufficient structural stability under both dry and swollen conditions. This combined liquid accessibility and network integrity establishes the structural foundation for the superior SA adsorption and sustained-release behavior discussed in the following sections.

### 2.2. Structural Optimization of TBA-Treated Aerogels

**Figure 2:**
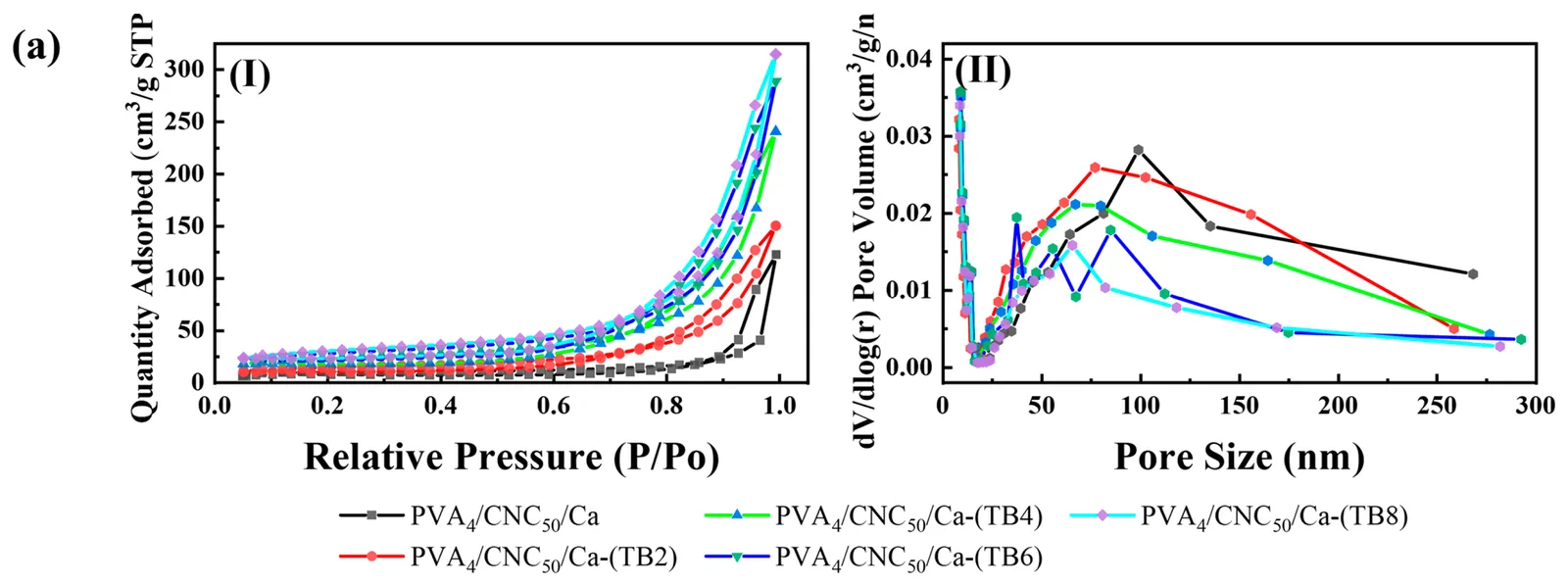

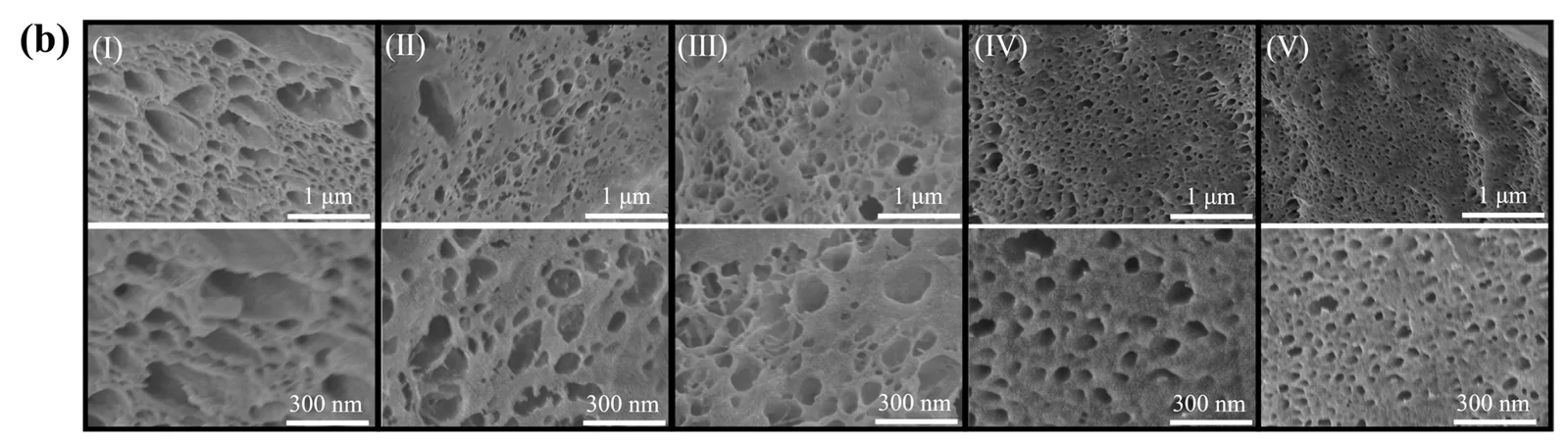
(**a**) Nitrogen adsorption–desorption isotherms (**I**) and pore size distribution curves (**II**) of PVA_4_/CNC_50_/Ca aerogels after TBA solvent exchange for different durations; (**b**) Low-magnification and high-magnification SEM images of the corresponding aerogels: (**I**) untreated, (**II**) TBA2, (**III**) TBA4, (**IV**) TBA6, and (**V**) TBA8. The scale bars are 1 μm for the low-magnification images and 300 nm for the high-magnification images. TBA2, TBA4, TBA6, and TBA8 denote TBA-treated aerogels with solvent-exchange durations of 2, 4, 6, and 8 h, respectively.

Figure 2 and Table 1 present the effect of TBA solvent exchange on the porous architecture of PVA_4_/CNC_50_/Ca aerogels. The untreated aerogel exhibited an irregular porous morphology with partially collapsed pore walls and locally compacted regions, reflecting that direct freeze-drying from water provoked substantial structural deformation. This collapse primarily arises from ice-crystal growth, crystallization-induced stress, and solute redistribution during the freezing step, which disturb the hydrogel skeleton and reduce the preservation of the internal porous network after sublimation [19,34]. In contrast, the TBA-treated aerogels displayed more clearly defined pore walls and a more open cellular structure. As the solvent-exchange duration increased from TBA2 to TBA6, the pore structure became progressively more uniform and continuous. Hence, TBA exchange effectively reduced drying-induced pore collapse and promoted the retention of the aerogel framework [23,26]. However, when the exchange duration was further extended to TBA8, the observed structural and physicochemical trends were consistent with a finer and potentially more compact pore structure. This observation suggests that prolonged solvent exchange may promote local network contraction or pore-wall consolidation; however, this interpretation is indirect and should not be considered direct evidence of pore-wall densification.

The nitrogen adsorption–desorption results further confirm this duration-dependent structural evolution. The specific surface area remarkably increased from 33.11 m^2^·g^−1^ for the untreated aerogel to 96.02 m^2^·g^−1^ for TBA6, while the average pore diameter decreased from 94.54 to 66.12 nm. Concurrently, the porosity rose from 93.93% to 96.03%, reflecting that TBA6 possessed a better-preserved and more accessible porous network. To sum up, an appropriate TBA exchange duration simultaneously promoted pore preservation, pore refinement, and pore connectivity. The decrease in average pore diameter from the untreated sample to TBA6 did not indicate pore blockage; instead, together with the increased porosity and improved liquid uptake, it demonstrated the formation of a more refined but still connected porous network. Hence, TBA6 provided a larger effective liquid-accessible pore structure rather than merely a higher dry-state surface area.

In contrast, TBA8 exhibited a slightly higher specific surface area (101.05 m^2^·g^−1^) and a smaller average pore diameter (60.65 nm) than TBA6, while its porosity and liquid uptake decreased slightly. These results indicate that a higher dry-state BET surface area does not necessarily correspond to greater liquid-phase pore accessibility. Because these aerogels are predominantly macroporous, nitrogen adsorption measurements may not fully represent the pore volume and connectivity accessible to SA under hydrated conditions. Therefore, adsorption performance should be considered in relation to pore connectivity and liquid accessibility rather than BET surface area alone [35].

The combined trends in pore diameter, porosity, density, and liquid uptake further suggest that prolonged TBA exchange may produce a finer and potentially more compact network. Previous studies have shown that solvent exchange can influence nanocellulose assembly, shrinkage, pore development, and final aerogel structure [36,37,38]. Accordingly, prolonged TBA exposure may promote local network rearrangement or pore-wall consolidation, potentially reducing the accessibility of some internal pore channels. However, this interpretation is based on indirect structure–property evidence and should not be considered direct proof of pore-wall densification. Overall, the SEM, BET, porosity, and liquid-uptake results indicate that TBA6 provided the most favorable balance between pore refinement, pore openness, and network stability among the investigated samples, which is beneficial for maintaining accessible adsorption sites and continuous diffusion pathways for drug loading and release [22,39].

Because direct diffusion coefficient or permeability measurements were not performed, the proposed changes in pore accessibility should be regarded as a structure–performance interpretation based on the available indirect evidence [23].

It should also be noted that the present study was conducted using a fixed CNC-containing PVA/CNCs/Ca^2+^ composition and did not include a CNC-free control. Therefore, the observed changes in pore structure and related transport properties demonstrate the effect of TBA solvent-exchange duration within the PVA/CNCs/Ca^2+^ composite system, but do not independently establish the contribution of CNC incorporation. Although CNCs may contribute to network reinforcement, pore development, and mass transport, their specific contribution cannot be separated from the overall composite-network response based on the current experimental design. Future studies incorporating CNC-free controls will be necessary to distinguish the individual and potentially coupled effects of CNC incorporation and solvent-exchange duration.

### 2.3. Pore Accessibility-Governed SA Adsorption and Diffusion-Regulated Release

**Figure 3:**
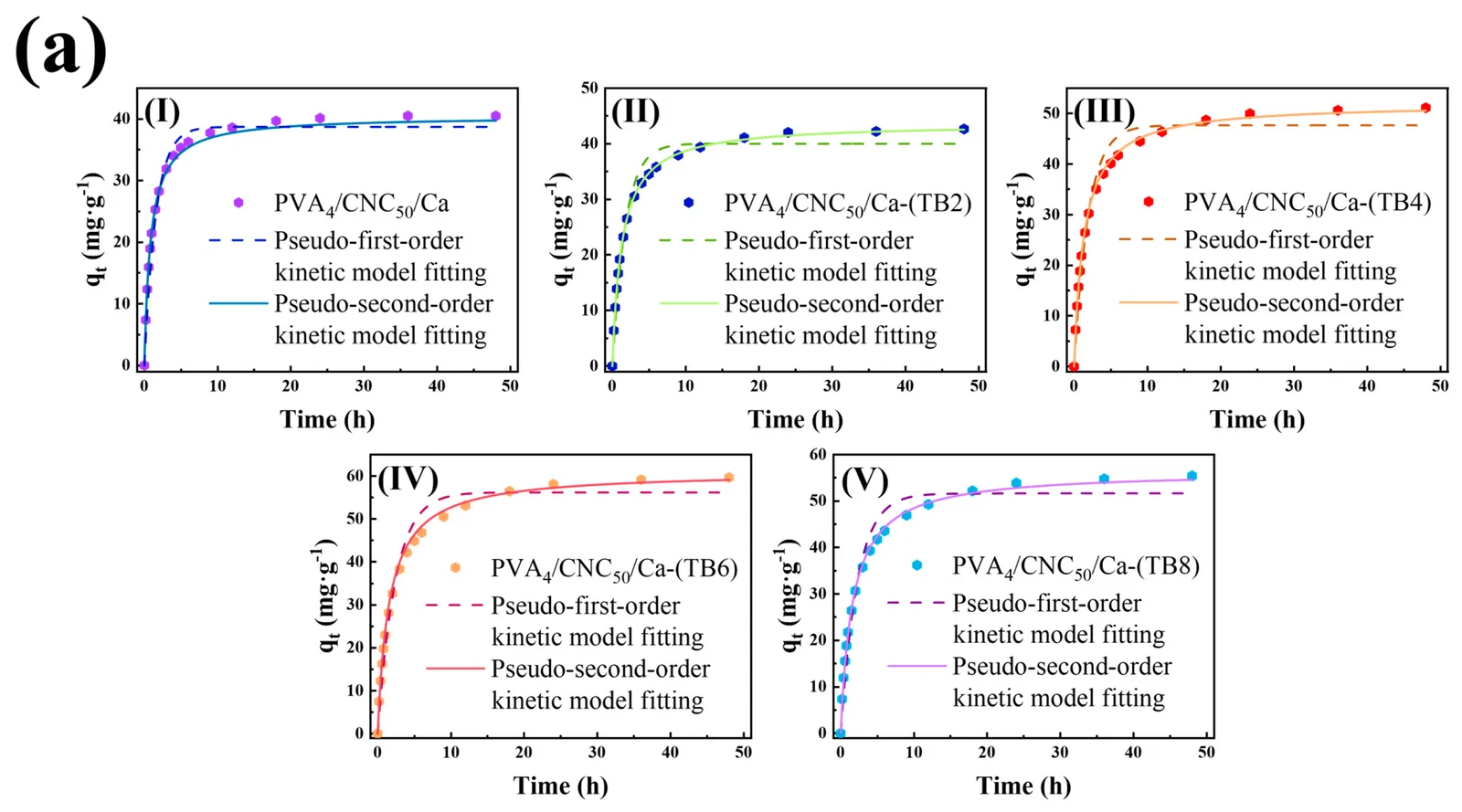

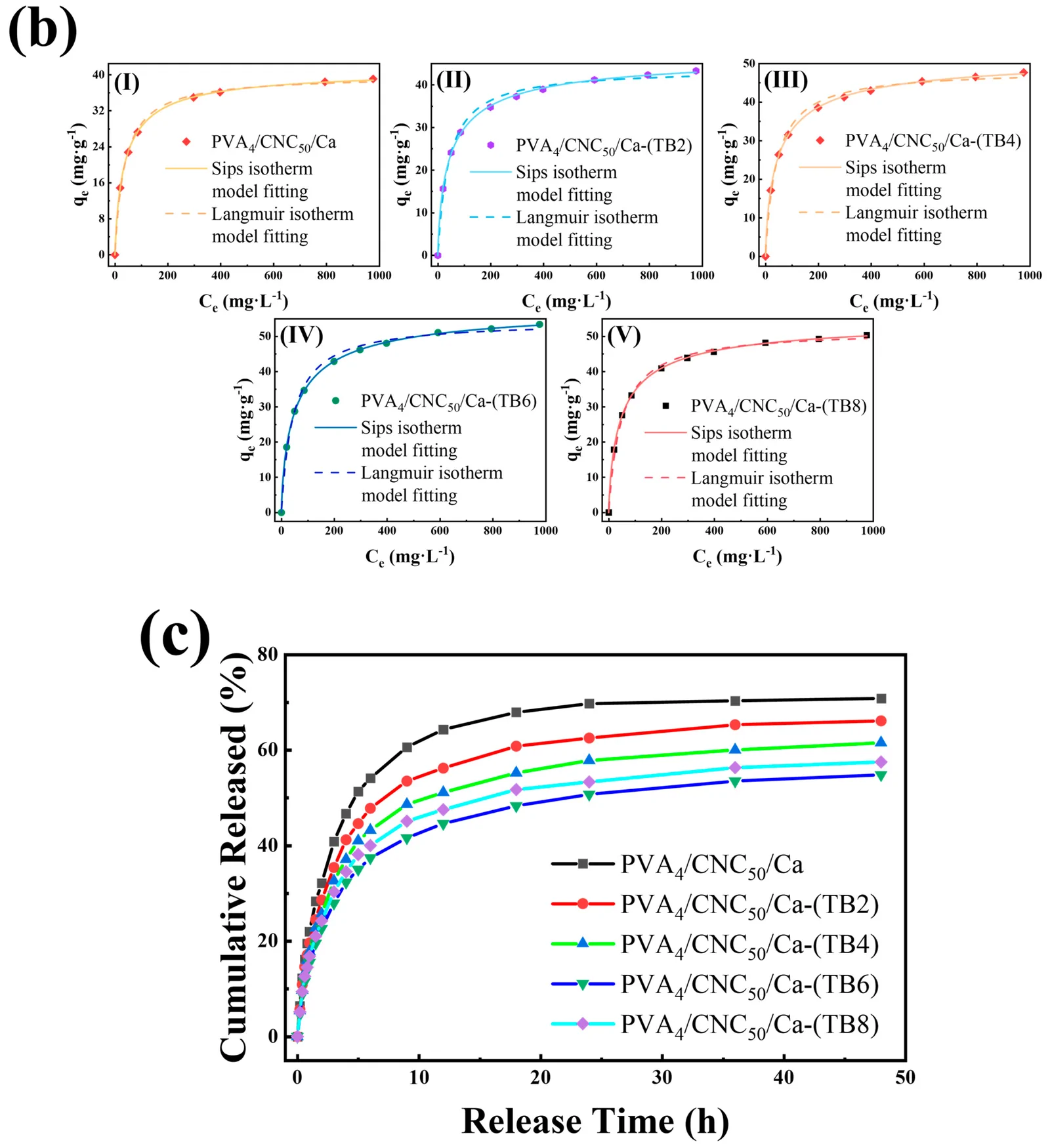
Effects of TBA solvent exchange on SA release and adsorption in PVA_4_/CNC_50_/Ca aerogels: (**a**) adsorption kinetic fitting curves; (**b**) adsorption isotherm fitting curves; (**c**) cumulative release profiles. TBA2, TBA4, TBA6, and TBA8 denote TBA-treated aerogels with solvent-exchange durations of 2, 4, 6, and 8 h, respectively.

The SA adsorption kinetic curves, adsorption isotherms, and release profiles are offered in Figure 3 and Table 2, Table 3, Table 4 and Table 5. For the purpose of following the experimental sequence and establishing drug loading before the interpretation of drug release, the adsorption kinetics and equilibrium adsorption behavior are discussed first, followed by analysis of the subsequent release profiles.

The adsorption kinetic curves in Figure 3a reveal that SA uptake occurred rapidly at the beginning and then gradually approached equilibrium. The experimentally measured equilibrium adsorption capacity increased progressively from 40.91 mg·g^−1^ for the untreated PVA4/CNC50/Ca aerogel to 43.63, 50.45, and 61.18 mg·g^−1^ for TBA2, TBA4, and TBA6, respectively. The equilibrium adsorption capacity decreased to 55.47 mg·g^−1^ as the solvent-exchange duration was further increased to TBA8. Thus, TBA6 demonstrated the highest experimentally measured SA uptake among the investigated samples.

The pseudo-second-order model gave fitted equilibrium adsorption capacities of 42.32, 45.11, 51.97, 61.89, and 56.42 mg·g^−1^ for the untreated, TBA2, TBA4, TBA6, and TBA8 aerogels, respectively. These fitted values were close to the corresponding experimental values. Since this trend is in good agreement with the structural analysis in Section 2.1 and Section 2.2, TBA6 provided more effective liquid-accessible adsorption sites and better pore connectivity than the untreated and TBA8 samples. Additionally, the pseudo-second-order model demonstrated consistently high coefficients of determination, with R^2^ values ranging from 0.989 to 0.996, compared with 0.934–0.972 for the pseudo-first-order model. Thus, the pseudo-second-order model provided the better empirical description of SA uptake for the investigated aerogels, reflecting that the adsorption rate was more closely related to the availability of accessible adsorption sites and the internal transport process within the porous network. Nevertheless, pseudo-second-order fitting should not be interpreted as direct proof of chemisorption or covalent bond formation [40,41]. This kinetic model could also describe adsorption controlled by a combination of accessible-site availability, liquid penetration, pore diffusion, and weak solute-matrix interactions. In the present system, the improved SA uptake after TBA treatment stems from increased liquid accessibility of the internal pore network and a larger number of accessible adsorption and storage domains.

The equilibrium adsorption results in Figure 3b were analyzed by the Langmuir and Sips isotherm models. The Sips model yielded R^2^ values of 0.997–0.999, which were consistently higher than the Langmuir-model values of 0.990–0.991. The Sips maximum adsorption capacity rose from 41.34 mg·g^−1^ for the untreated aerogel to 59.35 mg·g^−1^ for TBA6 and then dropped to 55.33 mg·g^−1^ for TBA8 (Table 3). The higher goodness of fit of the Sips model indicates that SA adsorption occurred on energetically or structurally heterogeneous adsorption domains rather than on an ideal homogeneous monolayer surface.

SA adsorption is likely governed by multiple weak interactions within the PVA/CNCs/Ca^2+^ network. The carboxyl and phenolic hydroxyl groups of SA can form hydrogen bonds with hydroxyl groups on PVA and CNCs, as well as with carboxyl groups on carboxylate CNCs. In contrast, π–π interactions are not a dominant adsorption mechanism, considering that the PVA/CNCs network does not contain obvious aromatic domains. Under aqueous conditions, the carboxyl group of SA may partially dissociate into salicylate, and Ca^2+^ may further facilitate adsorption through ionic bridging or coordination between carboxylate groups on CNCs and SA. Nevertheless, these interactions should be regarded as possible contributing factors in the absence of direct spectroscopic evidence. Therefore, SA adsorption in this system is more reasonably attributed to the combined effects of hydrogen bonding, possible Ca^2+^-mediated ionic interactions, physical confinement, pore accessibility, heterogeneous adsorption sites, and diffusion regulation within the aerogel matrix.

Notably, the increase in SA adsorption capacity was much smaller than the increase in BET surface area from 33.11 to 96.02 m^2^·g^−1^, highlighting that SA uptake was not governed by total dry-state surface area alone. Therefore, SA storage in the aerogel should be interpreted as a combined process involving penetration of SA solution into connected pores, physical confinement within the porous network, adsorption onto liquid-accessible internal surfaces, and weak interactions with the PVA/CNCs/Ca^2+^ network.

After the SA uptake behavior had been established, the loaded aerogels were evaluated for release in PBS. The untreated aerogel released SA more rapidly, whereas the TBA-treated samples demonstrated reduced release rates and more sustained release profiles (Figure 3c). The release data were fitted using first-order, zero-order, Higuchi, and Korsmeyer–Peppas models to statistically compare the release models (Table 4). The fitting performance was compared using the R^2^, root mean square error (RMSE), and Akaike information criterion (AIC) values summarized in Table 5. Among these empirical models, the Higuchi and Korsmeyer-Peppas models presented relatively higher R^2^ values and lower RMSE and AIC values, supporting a diffusion-regulated release tendency [42,43,44]. Similarly, network-dependent drug loading and diffusion have been reported in microporous hydrogel systems, where variations in polymer-network composition resulted in tunable drug-loading and release profiles [45]. Particularly, TBA6 displayed a lower Korsmeyer-Peppas release constant, decreasing from 26.10 to 18.14%·h^−1/2^, and its Higuchi constant dropped from 25.61 to 18.67, indicating a slower diffusion-regulated release process. Notably, no specific two-stage release model was applied. Consequently, the release profiles are discussed as an initially faster release followed by a gradually reduced release rate, instead of as a separately validated two-stage mechanism.

Drug release kinetics from porous matrices are strongly dependent on both internal pore architecture and external experimental parameters, including specimen dimensions, medium volume, and hydrodynamic stirring conditions. In this study, standardized thin cylindrical aerogel discs (10 mm diameter × 1.5 mm thickness) were evaluated in a bulk release volume (250 mL) under continuous gentle stirring (100 rpm). The thin-disc geometry minimizes the influence of sample thickness on external mass transfer and provides a simplified diffusion pathway, consistent with the assumptions commonly used for empirical Higuchi and Korsmeyer-Peppas analyses. Furthermore, thermodynamic saturation back-pressure is eliminated by maintaining strict sink conditions to ensure the constant driving force across the boundary. Concurrently, continuous agitation minimizes the boundary-layer mass-transfer resistance at the aerogel surface. Under these standardized hydrodynamic and geometric conditions, the observed reduction in Korsmeyer-Peppas and Higuchi release constants from untreated to TBA6 aerogels suggests that the reduced release constants are primarily associated with enhanced internal diffusion resistance and structural regulation of the hydrated matrix, rather than being dominated by external boundary-layer resistance.

To place the present release behavior in the context of the aerogel literature, Bang et al. [46] reported dual-stage release from polyurea-crosslinked dysprosia/silica and ordered mesoporous silica aerogels, featuring an initial rapid phase followed by slower release attributed to strong drug-skeleton interactions. Their systems combine rigid, non-swelling matrices, mesoscale confinement, and strong drug–skeleton binding, enabling distinct kinetic populations. In contrast, the present PVA/CNCs/Ca^2+^ aerogels are predominantly macroporous (Figure 2a), hydrate, and swell in PBS, while retaining SA through weak, reversible hydrogen bonding and possible Ca^2+^ bridging. Accordingly, Figure 3c demonstrates a monotonic decrease in release rate toward a plateau, without a secondary inflection or resolvable second phase. Single Higuchi and Korsmeyer-Peppas models describe the full profiles (Table 4 and Table 5). Hence, the release is characterized as a single-stage, matrix-diffusion-controlled process instead of a two-stage model. Additionally, 30–45% of loaded SA remained after 48 h, consistent with the weak residual Raman signal (Section 2.4). Nevertheless, this is interpreted as an approach toward partition equilibrium within the hydrated matrix but not as evidence of a distinct storage domain. Longer-duration release measurements would be required to distinguish these possibilities and avoid an unsupported assignment of multistage drug storage or release in the present system.

The adsorption and release results are consistent with the structural differences identified in Section 2.1 and Section 2.2. TBA6 exhibited the highest SA uptake together with lower release constants, indicating that its optimized porous architecture facilitated SA penetration and loading while maintaining sufficient diffusion resistance for sustained release. The lower adsorption capacity of TBA8 despite its higher BET surface area further supports the importance of effective liquid-accessible pore connectivity rather than dry-state surface area alone. Because direct diffusion coefficient or permeability measurements were not performed, this structure–performance relationship should be regarded as an interpretation based on the combined structural, adsorption, and release data.

SA was employed in this study as a small-molecule model drug to evaluate the relationship between pore accessibility and adsorption/release behavior. However, the present results should not be directly generalized to therapeutic molecules with substantially different molecular sizes or physicochemical properties. Larger molecules may experience greater steric restriction and diffusion resistance within the aerogel network, which could reduce penetration into less accessible pores and alter both loading capacity and release kinetics. In addition, drug–matrix interactions may vary with molecular structure and functionality. Therefore, although the duration-dependent regulation of pore accessibility identified here may provide a general design principle, the optimal pore architecture and solvent-exchange conditions are expected to depend on the properties of the therapeutic molecule. Further studies using drugs with different molecular sizes and clinically relevant properties are needed to establish the broader applicability of this aerogel platform.

### 2.4. Raman Visualization of SA Distribution in TBA6

**Figure 4:**
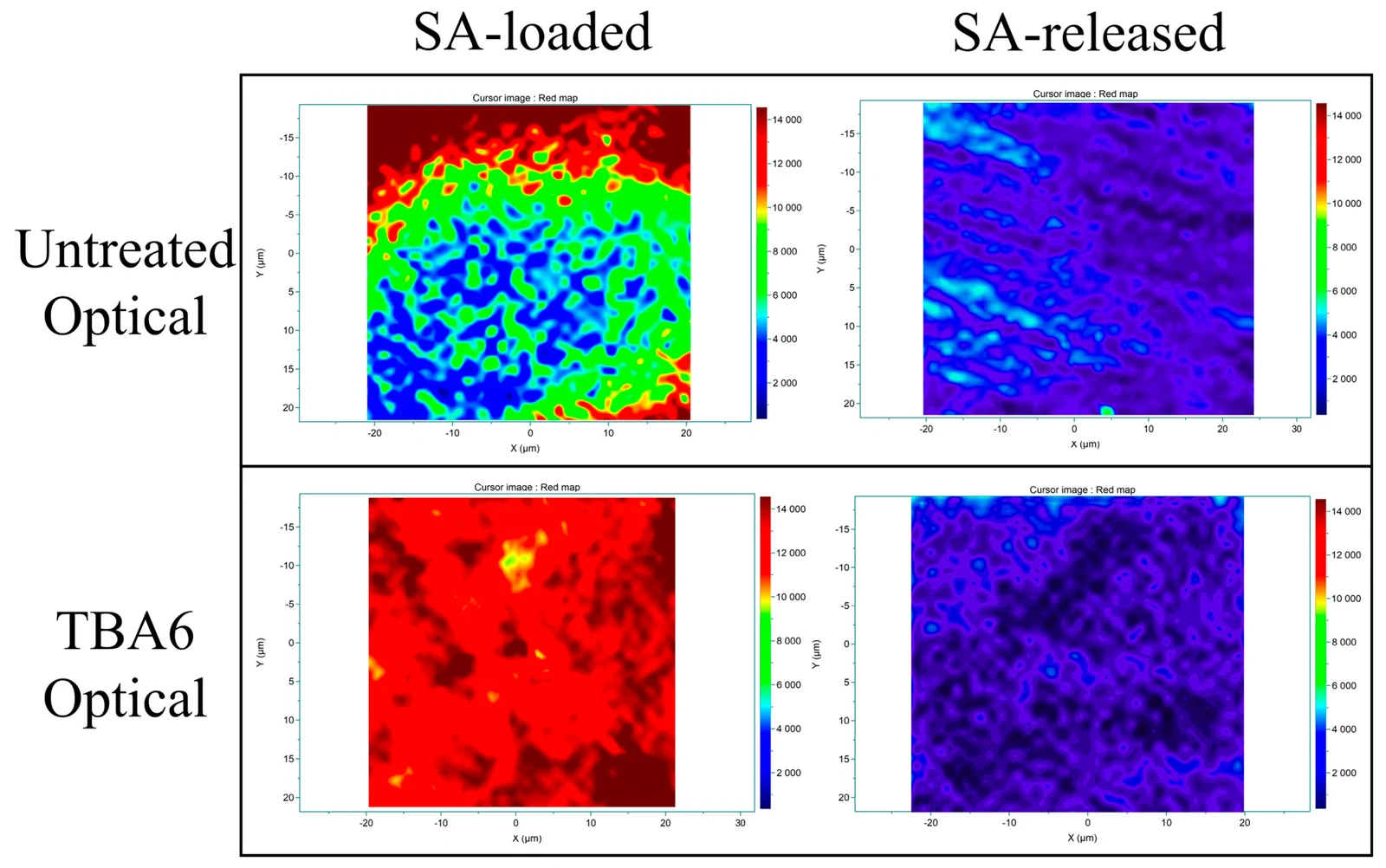
Optical images and confocal Raman maps of the microscale spatial distribution of SA in the middle regions of untreated and TBA6-treated aerogels. The color scale represents the relative Raman signal intensity of SA, with higher intensity indicating a stronger SA signal.

Confocal Raman imaging was employed to visualize the microscale spatial distribution of SA within the aerogels (Figure 4). TBA6 demonstrated a more uniform SA signal after loading than the untreated aerogel, reflecting improved drug penetration and adsorption homogeneity. After release, the weakened but still detectable SA signal exhibited gradual diffusion from the internal pores, consistent with the sustained-release profiles. Additionally, Confocal Raman mapping was adopted to visualize the spatial distribution of SA in the untreated and TBA6-treated aerogels before and after release [47,48]. As observed from Figure 4, Raman maps were collected from the middle region of the aerogel sections to compare drug distribution within the internal matrix. Before release, the untreated aerogel exhibited relatively uneven SA signals, with locally concentrated regions and less homogeneous intensity distribution. In other words, the partially collapsed pore structure limited SA penetration and induced preferential accumulation in more accessible regions. In contrast, TBA6 presented more continuous and uniform SA signals within the mapped region, verifying the improved SA penetration and more homogeneous drug loading in the aerogel matrix. Following release, the Raman intensity decreased significantly for both samples, confirming SA diffusion into the release medium. Nevertheless, weak residual SA signals were still detectable in TBA6. Thus, part of the SA was retained within the internal porous network and released gradually rather than being rapidly depleted from the external surface.

These Raman observations are in line with the adsorption and release results discussed in Section 2.3. The uneven SA distribution in the untreated aerogel suggests that its partially collapsed pore structure restricted solution penetration, leading to heterogeneous SA distribution with higher local concentration in relatively accessible regions [49,50].

Such non-uniform drug distribution is consistent with its lower adsorption capacity and faster release behavior. In contrast, the optimized porous network facilitated more homogeneous SA infiltration and distribution throughout the aerogel matrix, resulting in improved drug-loading uniformity, as revealed by the more homogeneous SA signal observed in TBA6. Moreover, the residual Raman signal after release suggests that SA was not solely associated with the external surface. It remained distributed within the hydrated porous matrix and was gradually released during diffusion-controlled transport.

Together with the pore structure, liquid uptake, adsorption, and release results, the Raman maps (Figure 4) visually verify the proposed structure–performance relationship. Notably, Raman mapping was performed over a 20–100 μm field of view and therefore did not directly resolve SA localization within individual nanoscale pores observed in SEM images. Instead, it reflects the relative uniformity of SA distribution across the aerogel section, contributing to evaluating whether the optimized pore network facilitates more homogeneous drug penetration and retention at the microscale. Nevertheless, direct measurements of diffusion coefficients or permeability would be valuable for further validating the role of pore accessibility in mass transport. These measurements will be considered in future studies. The more homogeneous SA signal in TBA6 reflects improved SA penetration and distribution at the microscale, in good agreement with its higher adsorption capacity and slower release behavior. TBA solvent exchange curtailed drying-induced pore collapse and generated an aerogel network with improved pore accessibility and sufficient wet-state stability [22,32,51]. For TBA6, this balanced structure allowed SA molecules to diffuse into the internal pores during loading, while the regulated pore structure increased resistance to molecular transport during release. Hence, the sustained-release behavior of TBA6 is essentially governed by the coordinated effects of pore accessibility, network stability, liquid uptake, and diffusion-pathway regulation, rather than by strong chemical interactions [17,52]. To sum up, TBA6 exhibited the best overall balance between SA adsorption and sustained-release performance among the investigated aerogels.

## 3. Conclusions

TBA solvent exchange effectively regulated the porous architecture of PVA_4_/CNC_50_/Ca aerogels and improved their performance as SA delivery carriers. Among the investigated samples, TBA6 achieved the most favorable balance between pore openness, liquid-accessible pore connectivity, and network stability. Its specific surface area increased from 33.11 to 96.02 m^2^·g^−1^, while the experimentally measured equilibrium SA adsorption capacity rose from 40.91 to 61.18 mg·g^−1^. Simultaneously, the Korsmeyer-Peppas release constant decreased from 25.070 to 17.283%·h^−1/2^, and the Higuchi release constant dropped from 18.148 to 12.751, reflecting a slower diffusion-regulated release process. Together with the SEM, BET, porosity, liquid-uptake, adsorption, release-kinetic, and Raman mapping results, these findings revealed that the improved SA delivery performance was governed by the coordinated regulation of pore preservation, effective pore accessibility, drug distribution, and diffusion resistance. Additionally, Confocal Raman imaging verified that TBA6 promoted a more homogeneous SA distribution within the aerogel matrix and enabled gradual release from the internal porous network. To sum up, the improvement in SA delivery was concurrently governed by increased surface area, the coordinated regulation of pore preservation, pore accessibility, network stability, and diffusion resistance. Overall, duration-controlled TBA solvent exchange provides an effective pore-engineering strategy for optimizing PVA-based composite aerogels for drug-loading and sustained-release applications. In the future, the broader applicability and mechanistic basis of this aerogel platform should be clarified through CNC-free control systems, direct diffusion or permeability measurements, evaluation of CNC size and distribution effects, and release assessment under more application-relevant biological conditions.

## 4. Materials and Methods

### 4.1. Materials

PVA (MW 89–98 kDa, 99% hydrolyzed) was obtained from Shanghai Yingjia Industrial Development Co., Ltd. (Shanghai, China). SA was purchased from Tianjin Kermel Chemical Reagent Co., Ltd. (Tianjin, China). Sodium chloride (NaCl, ≥99.5%) and Calcium chloride (CaCl_2_, ≥99.5%) were procured from Shanghai Aladdin Biochemical Technology Co., Ltd. (Shanghai, China). CNCs were synthesized in our laboratory from ground hybrid poplar particles using ammonium persulfate oxidation [21], following previously described methods [53]. Their characteristics are detailed in Figure S1. Their dimensions were not remeasured in the present study but were taken from our previously published characterization of CNCs prepared using the same procedure [54].

### 4.2. Preparation of Composite Aerogels

**Figure 5:**
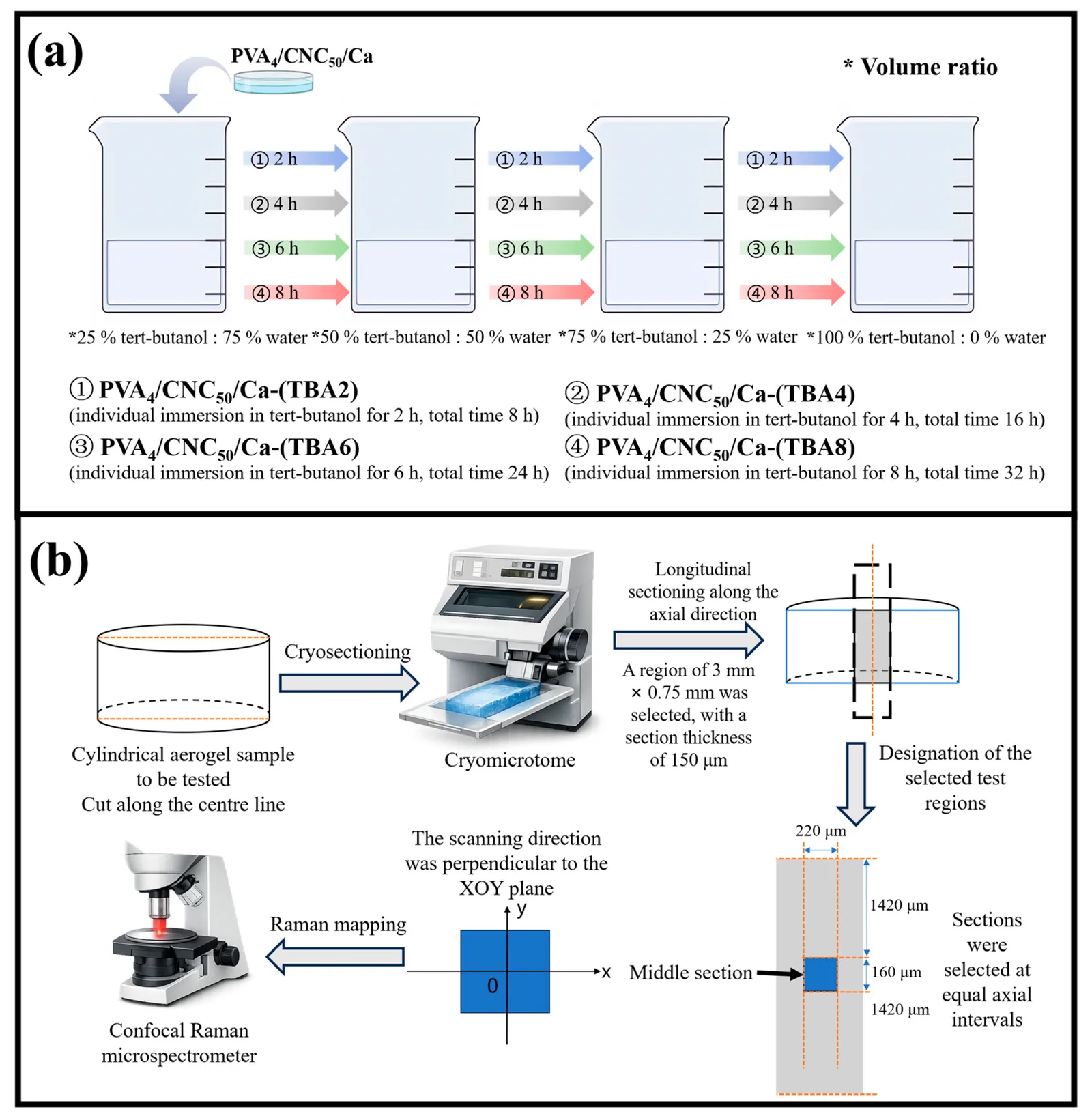
Schematic illustrations of (**a**) TBA solvent exchange and (**b**) confocal Raman mapping of aerogel sections.

PVA/CNCs/Ca^2+^ composite aerogels were prepared by physical/ionic crosslinking and freeze-drying. PVA (4 wt%) was dissolved in deionized water at 95 °C. CNCs (50 wt% relative to PVA) were ultrasonically dispersed in water and added to the PVA solution. Subsequently, CaCl_2_ solution (1 wt%) was added dropwise. For TBA treatment, the hydrogels were immersed successively in aqueous TBA solutions of 25%, 50%, 75%, and 100% for different times before freeze-drying. The treated samples were denoted as PVA_4_/CNC_50_/Ca-(TBAx), where x represents the soaking time (h) at each gradient. The untreated samples were denoted as PVA_4_/CNC_50_/Ca. TBA2, TBA4, TBA6, and TBA8 signify 2, 4, 6, and 8 h of immersion at each concentration step, corresponding to total solvent-exchange times of 8, 16, 24, and 32 h, respectively. The mixture was cast into a mold, frozen in LN, and then freeze-dried. The solvent exchange procedure is illustrated in Figure 5a.

### 4.3. Characterization of Composite Aerogels

#### 4.3.1. Density and Gravimetric PBS Uptake Test

Each aerogel sample was cut into a uniform shape and weighed accurately. Then, the samples were immersed in PBS (pH 7.4) for 6 h. Their weights were measured at predetermined time intervals. The gravimetric PBS uptake ratio (S) of the samples was calculated by:

(1) $$S=\frac{W_{t}-W_{0}}{W_{0}}\times 100\%$$

where W_0_ denotes the initial dry weight of the freeze-dried aerogel sample, and W_t_ signifies the weight of the sample after PBS uptake at time t. This parameter represents gravimetric liquid uptake rather than direct volumetric swelling.

The apparent porosity of the freeze-dried PVA/CNCs/Ca^2+^ composite aerogel samples was determined by the isopropanol immersion method. Aerogel samples from each group were cut into circular discs with a diameter of 10 mm and a thickness of 1.5 mm, with their dry weight (W_0_) being recorded. Afterward, the samples were completely immersed in isopropanol and subjected to vacuum treatment for 5 min to facilitate the full penetration of isopropanol into the pores of the aerogels. Following removal, the residual solvent on the sample surface was gently blotted with filter paper, and the saturated weight (W_1_) was measured immediately. The porosity (P) of the samples was calculated by:

(2) $$P=\frac{W_{1}-W_{0}}{\rho V}\times 100\%$$

where *ρ* represents the density of isopropanol (0.7855 g/mL), and V indicates the volume of the sample, calculated by the formula for the volume of a cylinder.

#### 4.3.2. Structural Characterization

Nitrogen adsorption–desorption measurements were conducted by a Micromeritics ASAP 2020 analyzer (ASAP 2020, Norcross, GA, USA) to determine the specific surface area and pore characteristics. Before analysis, samples (0.1–0.2 g) were degassed under vacuum at 60 °C for 12 h. The specific surface area was calculated by the BET method. The pore volume and size distribution were derived from the BJH model. The microstructure of the aerogels was examined by scanning electron microscopy (SEM, JSM-7800F, JEOL Ltd., Tokyo, Japan).

#### 4.3.3. Adsorption Experiments: Adsorption Kinetics

SA adsorption kinetics onto aerogels were investigated by batch adsorption methods. Aerogel samples (100 mg) were immersed in 250 mL of SA solution (2000 mg·L^−1^) under gentle stirring. Aliquots were collected at predetermined intervals (0, 0.25, 0.5, 0.75, 1, 2, 3, and 6 h) for analysis. Preliminary experiments suggested that equilibrium was reached within 6 h. Residual SA concentration was determined spectrophotometrically at the maximum absorption wavelength (λ_max_). Adsorption capacities were calculated based on calibration curves.

Kinetic data were fitted by the pseudo-first-order and pseudo-second-order kinetic models to describe the adsorption behavior at the solid–liquid interface.

The Pseudo-first-order model is expressed as:

(3) $$q_{t}=q_{e}\left(1-e^{-k_{1}\cdot t}\right)$$

where q_t_ denotes the adsorption capacity at time t, q_e_ signifies the equilibrium adsorption capacity, and k_1_ represents the first-order adsorption rate constant.

The Pseudo-second-order model is expressed as:

(4) $$\frac{dq_{t}}{dt}=k_{2}{\left(q_{e}-q_{t}\right)}^{2}$$

where q_t_ and q_e_ are as defined above, and k_2_ indicates the pseudo-second-order adsorption rate constant.

#### 4.3.4. Adsorption Experiments: Adsorption Isotherms

Adsorption isotherms were obtained by equilibrating 100 mg of aerogel samples with SA solutions of varying concentrations (25–1000 mg·L^−1^) at 37 °C under continuous stirring at 100 rpm until equilibrium was reached. Residual SA concentrations were determined spectrophotometrically, and equilibrium adsorption capacities were calculated.

Equilibrium data were analyzed by the Langmuir and Sips isotherm models:

The Langmuir model is expressed as:

(5) $$q_{e}=\frac{q_{max}k_{L}C_{e}}{1+k_{L}C_{e}}$$

where q_max_ represents the maximum monolayer adsorption capacity, K_L_ denotes the Langmuir constant, and C_e_ indicates the equilibrium solute concentration.

The Sips isotherm model is expressed as:

(6) $$q_{e}=\frac{q_{max}{\left(K_{S}+C_{e}\right)}^{n}}{1+{\left(K_{S}+C_{e}\right)}^{n}}$$

where q_max_ and C_e_ are as defined above, K_S_ represents the Sips equilibrium constant, and n refers to the heterogeneity factor.

#### 4.3.5. In Vitro Drug Release

The calibration curve of SA in PBS was established by ultraviolet-visible (UV-vis) spectroscopy (UV-2802, Shimadzu, Kyoto, Japan). Aerogels with a diameter of 10 mm and a thickness of 1.5 mm were immersed in a supersaturated aqueous SA solution at 37 °C for 24 h. The absorbed solution volume was recorded, and SA concentration was quantified at 297 nm using a UV-Vis spectrophotometer (UV-2600, Shimadzu, Japan). The SA-loaded aerogels were then transferred into 250 mL PBS (pH 5) at 37 ± 0.5 °C. The release medium was agitated at 100 rpm using an orbital shaker. At predetermined intervals, 3 mL aliquots were withdrawn and immediately replaced with an equal volume of fresh pre-warmed PBS. Released SA concentration was measured spectrophotometrically at λ_max_ = 296 nm, and cumulative release percentages were calculated. All groups were evaluated using identical specimen geometry, medium volume, temperature, and agitation conditions to ensure direct comparability of the fitted release constants. The UV-vis standard curves and regression line for the SA are shown in Figure S2.

Data were fitted by the first-order and Korsmeyer–Peppas kinetic models to elucidate the release mechanism. Additionally, data from three independent measurements (n = 3) were fitted by first-order, zero-order, Higuchi, and Korsmeyer-Peppas kinetic models to evaluate the release behavior. The fitting quality of different models was compared with respect to R^2^, RMSE, and AIC:

The first-order model is expressed as:

(7) $$C_{t}=C_{0}\cdot e^{-k_{e}\cdot t}$$

where C_0_ denotes the initial drug concentration, C_t_ represents the remaining drug percentage at time t, and k_e_ indicates the first-order release rate constant.

#### 4.3.6. Tensile Strength

The elongation at break and elastic modulus of aerogels at different compositions before and after equilibrium-swelling were measured by a universal testing machine (TY-8000B, Tianjing, China). Dumbbell-shaped specimens (115 × 25 × 3 mm) were subjected to uniaxial tension at a crosshead speed of 20 mm·min^−1^ at 25 °C. Each reported value represents the mean of three independent measurements. The elastic modulus was determined from the slope of the initial linear region of the stress–strain curve.

#### 4.3.7. Confocal Raman Micro-Spectroscopy Test

The distribution of SA in the aerogels was analyzed by confocal Raman micro-spectroscopy (XploRA Plus, HORIBA, Kyoto, Japan). The aerogel loaded with SA and the aerogel released at least 72 h after SA release were first sectioned and then tested through confocal Raman spectroscopy. The frozen microtome (CM1850, Leica, Wetzlar, Germany) was used for sectioning. SA-loaded aerogels and aerogels after 72 h of release were longitudinally sectioned along the centerline using a cryomicrotome (CM1850, Leica, Germany). The section thickness was approximately 150 μm to obtain a relatively flat observation surface and reduce light-scattering interference. Representative regions were selected within an axial area of approximately 3 mm × 0.75 mm, with a central analysis region of approximately 220 μm × 160 μm. For microscale Raman mapping, local regions with a field of view of at least 40 μm × 40 μm were scanned in confocal mode to minimize interference from out-of-focus signals. The spatial distribution of SA was visualized based on the Raman intensity of its characteristic band, and the relative SA signal distributions before and after release were compared. The specific operation process of the confocal Raman spectrometer is illustrated in Figure 5b.

#### 4.3.8. Statistical Analysis

All quantitative data are presented as mean ± standard deviation (SD) from three independent measurements unless otherwise stated. Statistical analysis was performed by IBM SPSS Statistics 20 (International Business Machines Corporation, Amonk, NY, USA). Differences among groups were evaluated by one-way analysis of variance (ANOVA), and *p* < 0.05 indicates a statistically significant level. Release-kinetic fitting was performed by Origin 2026, and model fitting performance was compared with respect to R^2^, RMSE, and AIC values.

#### 4.3.9. Use of Generative AI Tools

ChatGPT (OpenAI, Version 5.6.0) was employed to assist in preparing schematic graphical elements in the graphical abstract and selected illustrative figures. All AI-assisted graphical content was revised, verified, and finalized by the authors to ensure scientific accuracy and consistency with the experimental results. No AI tool was used to generate or alter original experimental data, SEM images, Raman maps, or quantitative plots.

## Figures and Tables

**Table 1:** Pore-structure parameters of PVA_4_/CNC_50_/Ca composite aerogels with different TBA substitution times.

Samples	Specific Surface Area (m^2^·g^−1^)	Pore Capacity (cm^3^·g^−1^)	Porosity (%)	Pore Diameter (nm)
PVA_4_/CNC_50_/Ca	33.11	0.050	93.93	94.54
PVA_4_/CNC_50_/Ca-(TBA2)	59.08	0.038	95.26	93.42
PVA_4_/CNC_50_/Ca-(TBA4)	72.68	0.029	95.66	71.53
PVA_4_/CNC_50_/Ca-(TBA6)	96.02	0.019	96.03	66.12
PVA_4_/CNC_50_/Ca-(TBA8)	101.05	0.020	95.84	60.65

**Table 2:** Kinetic parameters for SA adsorption onto PVA_4_/CNC_50_/Ca composite aerogel samples derived from different kinetic models.

Samples	Experimental q (mg/g)	First-Order Kinetic Model			Linear Second-Order Kinetic		
		k_1_ (h^−1^)	q_e_ (mg·g^−1^)	R^2^ (−)	k_2_ (g·mg^−1^·h^−1^)	Fitted q_e_ (mg·g^−1^)	R^2^ (-)
PVA_4_/CNC_50_/Ca	40.91	0.636	38.71	0.934	0.0287	42.32	0.996
PVA_4_/CNC_50_/Ca-(TBA2)	43.63	0.567	39.99	0.972	0.0178	45.11	0.996
PVA_4_/CNC_50_/Ca-(TBA4)	50.45	0.515	47.63	0.967	0.0141	51.97	0.993
PVA_4_/CNC_50_/Ca-(TBA6)	61.18	0.418	56.18	0.963	0.0104	61.89	0.989
PVA_4_/CNC_50_/Ca-(TBA8)	55.47	0.446	51.61	0.965	0.0117	56.42	0.996

**Table 3:** Parameters derived from Sips and Langmuir adsorption isotherms for PVA_4_/CNC_50_/Ca composite aerogels with different TBA substitution times.

Samples	Sips Isotherm Model				Langmuir Isotherm Model		
	q_max_ (mg·g^−1^)	K_S_ (L^n^·mg^−n^)	*n* (−)	R^2^ (−)	q_max_ (mg·g^−1^)	*K_L_* (L·mg^−1^)	R^2^ (−)
PVA_4_/CNC_50_/Ca	41.34	0.0260	0.85	0.997	39.86	0.0270	0.990
PVA_4_/CNC_50_/Ca-(TB2)	44.66	0.0199	0.74	0.999	41.77	0.0244	0.990
PVA_4_/CNC_50_/Ca-(TB4)	51.49	0.0197	0.75	0.999	47.34	0.0234	0.991
PVA_4_/CNC_50_/Ca-(TB6)	59.35	0.0183	0.76	0.999	54.39	0.0224	0.990
PVA_4_/CNC_50_/Ca-(TB8)	55.33	0.0196	0.77	0.999	51.83	0.0209	0.991

**Table 4:** Release curves and fitting parameters of PVA_4_/CNC_50_/Ca composite aerogels with different TBA substitution times.

Samples	First-Order		Zero-Order		Higuchi		Korsmeyer-Peppas		
	*k_e_* (h^−1^)	R^2^ (−)	*k*_0_ (%·h^−1^)	R^2^ (−)	*k_H_* (−)	R^2^ (−)	*k_k-p_* (%·h^−1/2^)	*n* (−)	R^2^ (h^−n^)
PVA_4_/CNC_50_/Ca	0.120	0.739	4.315	0.102	18.148	0.873	25.070	0.360	0.951
PVA_4_/CNC_50_/Ca-(TBA2)	0.087	0.649	3.832	0.130	16.068	0.885	21.990	0.364	0.957
PVA_4_/CNC_50_/Ca-(TBA4)	0.070	0.590	3.502	0.142	14.666	0.890	19.999	0.366	0.960
PVA_4_/CNC_50_/Ca-(TBA6)	0.053	0.523	3.051	0.161	12.751	0.898	17.283	0.368	0.965
PVA_4_/CNC_50_/Ca-(TBA8)	0.060	0.537	3.252	0.129	13.630	0.887	18.683	0.363	0.961

**Table 5:** Goodness-of-fit statistics (RMSE and AIC) for kinetic models describing SA release from PVA_4_/CNC_50_/Ca composite aerogels with different TBA solvent-exchange durations.

Samples	First-Order			Zero-Order			Higuchi			Korsmeyer-Peppas		
	R^2^ (−)	RMSE (%)	AIC (−)	R^2^	RMSE (%)	AIC (−)	R^2^ (−)	RMSE (%)	AIC (−)	R^2^ (h^−n^)	RMSE (%)	AIC (−)
PVA_4_/CNC_50_/Ca	0.739	11.377	81.81	0.102	21.087	101.56	0.873	7.933	70.27	0.951	4.940	57.12
PVA_4_/CNC_50_/Ca-(TBA2)	0.649	11.669	82.62	0.130	18.377	97.16	0.885	6.692	64.83	0.957	4.089	51.06
PVA_4_/CNC_50_/Ca-(TBA4)	0.590	11.509	82.18	0.142	16.647	93.99	0.890	5.968	61.17	0.960	3.595	46.95
PVA_4_/CNC_50_/Ca-(TBA6)	0.523	10.788	80.11	0.161	14.302	89.13	0.898	4.986	55.41	0.965	2.924	40.33
PVA_4_/CNC_50_/Ca-(TBA8)	0.537	11.326	81.67	0.129	15.531	91.77	0.887	5.589	59.07	0.961	3.306	44.26

## Data Availability

The raw data supporting the conclusions of this article will be made available by the authors upon request.

## Supplementary Materials

The following supporting information can be downloaded at: https://www.mdpi.com/article/10.3390/gels12090791/s1, Figure S1. (a) Fourier-transform infrared (FT-IR) spectra, (b) X-ray diffraction pattern (XRD), and (c) thermogravimetric (TG) analysis curve of the CNCs prepared by ammonium persulfate oxidation. Figure S2. (a) UV-vis absorption spectra at different salicylic acid concentrations and (b) corresponding calibration curve with linear regression.

## Abbreviations

The following abbreviations are used in this manuscript:

BET	Brunauer Emmett Teller
BJH	Barrett-Joyner-Halenda
Ca^2+^	calcium ions
CaCl_2_	calcium chloride
CNCs	cellulose nanocrystal
FT-IR	Fourier-transform infrared
LN	liquid nitrogen
NaCl	sodium chloride
PBS	phosphate-buffered saline
PVA	poly (vinyl alcohol)
SEM	scanning electron microscopy
SA	salicylic acid
TBA	tert-butanol
TG	thermogravimetric
UV	ultraviolet
UV-Vis	Ultraviolet-visible spectroscopy
XRD	x-ray diffractometry
