# Supplementary material for: Engineering Pore Accessibility in PVA/Cellulose Nanocrystal/Ca2+ Composite Aerogels for Enhanced Drug Adsorption and Sustained Release
Source: Gels. 2026 Sep 1;12(9):791. doi: 10.3390/gels12090791 (PMC13606626; doi:10.3390/gels12090791)
Supplement: Supplementary file 1 [file gels-12-00791-s001.zip › gels-4443875-supplementary.pdf]

Supporting Information

Engineering Pore Accessibility in PVA/Cellulose Nanocrystal/Ca^{2+} Composite Aerogels for Enhanced Drug Adsorption and Sustained Release

Zixuan Zhang, Qianqian Wu and Hua Jiang *

College of Chemical Engineering, Nanjing Forestry University, Nanjing 210037, China

* Correspondence: jianghua@njfu.com.cn

The preparation of the CNCs and their properties:

The dried and ground hybrid poplar particles by ammonium persulfate oxidation was treated with toluene/ethanol and ethanol / NaOH sequentially, and then the treated suspension was filtrated. The solid residue was dried under vacuum for 24 h, and ground. Then, the dry solid residue was treated with 1 mol·L^{-1} APS solution at 70 ℃ for a given time, followed the centrifugation/washing, until the solution conductivity was closed to that of deionized water. All the resulting precipitate was ultrasonicated in deionized water to form the uniform and translucent CNC colloid suspension. The CNCs used in this work are carboxylate CNCs.

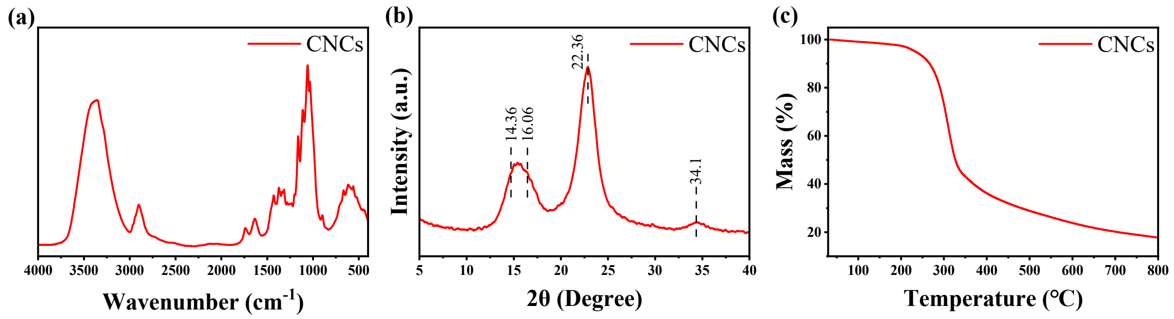

Figure S1. (a) Fourier-transform infrared (FT-IR) spectra, (b) X-ray diffraction pattern (XRD), and (c) thermogravimetric (TG) analysis curve of the CNCs prepared by ammonium persulfate oxidation.

The absorption peak and standard curve of SA:

The calibration curve of SA in phosphate-buffered saline (PBS) was established by UV-vis spectroscopy (UV-2802, Shimadzu, Japan).

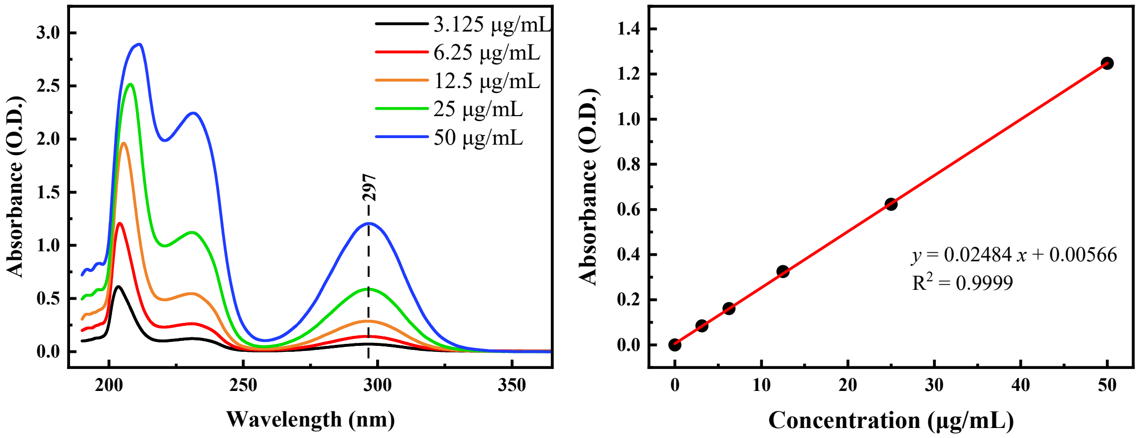

Figure S2. (a) UV-vis absorption spectra at different salicylic acid concentrations and (b) corresponding calibration curve with linear regression.
